# Repository of MRI-derived models of the breast with single and multiple benign and malignant tumors for microwave imaging research

**DOI:** 10.1371/journal.pone.0302974

**Published:** 2024-05-17

**Authors:** Ana C. Pelicano, Maria C. T. Gonçalves, Tiago Castela, M. Lurdes Orvalho, Nuno A. M. Araújo, Emily Porter, Raquel C. Conceição, Daniela M. Godinho

**Affiliations:** 1 Instituto de Biofísica e Engenharia Biomédica, Faculdade de Ciências, Universidade de Lisboa, Lisbon, Portugal; 2 Departamento de Física, Faculdade de Ciências, Universidade de Lisboa, Lisbon, Portugal; 3 Departamento de Radiologia, Hospital da Luz Lisboa, Luz Saúde, Lisboa, Portugal; 4 Centro de Física Teórica e Computacional, Faculdade de Ciências, Universidade de Lisboa, Lisbon, Portugal; 5 Chandra Family Department of Electrical and Computer Engineering, The University of Texas at Austin, Austin, TX, United States of Ameirca; 6 Department of Biomedical Engineering, McGill University, Montréal, Canada; Information Technology University, PAKISTAN

## Abstract

The diagnosis of breast cancer through MicroWave Imaging (MWI) technology has been extensively researched over the past few decades. However, continuous improvements to systems are needed to achieve clinical viability. To this end, the numerical models employed in simulation studies need to be diversified, anatomically accurate, and also representative of the cases in clinical settings. Hence, we have created the first open-access repository of 3D anatomically accurate numerical models of the breast, derived from 3.0T Magnetic Resonance Images (MRI) of benign breast disease and breast cancer patients. The models include normal breast tissues (fat, fibroglandular, skin, and muscle tissues), and benign and cancerous breast tumors. The repository contains easily reconfigurable models which can be tumor-free or contain single or multiple tumors, allowing complex and realistic test scenarios needed for feasibility and performance assessment of MWI devices prior to experimental and clinical testing. It also includes an executable file which enables researchers to generate models incorporating the dielectric properties of breast tissues at a chosen frequency ranging from 3 to 10 GHz, thereby ensuring compatibility with a wide spectrum of research requirements and stages of development for any breast MWI prototype system. Currently, our dataset comprises MRI scans of 55 patients, but new exams will be continuously added.

## Introduction

MWI has been studied in the past decades for breast cancer screening and diagnosis [1–3]. Since then, new and improved systems continue to emerge with the purpose of bringing breast MWI technology closer to clinical settings [4–19]. Besides breast cancer detection, MWI has also shown potential in other areas such as breast cancer staging through axillary imaging [20], brain stroke detection [21], and bone health monitoring [22].

Anatomically accurate breast models are imperative for the development of breast MWI diagnostic systems, as well as microwave hyperthermia and ablation therapeutic devices [23, 24], as they allow systems to be designed, tested, and validated under conditions representative of patients and clinical examinations. Hence, numerical models of the breast must evolve and become increasingly more realistic, accurately portraying the complex structures of all normal, benign, and malignant tissues. MRI images have been largely used as the basis for the construction of anatomically realistic breast models [25–30]. Additionally, data extracted from these exams, such as voxel intensity, has been used to assign dielectric properties of biological tissues measured in large-scale patient studies [31, 32] to individual models through different strategies [26–28]. This results in the creation of dielectric property maps for breast tissues at microwave frequencies. The estimation of the dielectric properties of tissues using MRI images have also been studied before. To the best of the authors’ knowledge, three different approaches that have been studied: one focusing on the acquisition of MRI sequences that can be used to determine the water content distribution of tissues, followed by the estimation of the dielectric properties of tissues based on this information [33, 34]; one concerning Electric Properties Tomography (EPT), which is based on B1 mapping and the estimation of tissues dielectric properties at the Larmor frequency [35–37], which can go up to 300 MHz; and finally, a recent strategy described in [38, 39] which combines the first two.

Most breast MWI research studies report highly accurate breast models concerning healthy breast tissues but oversimplified models of benign and malignant breast tumors [40–43]. These models are oftentimes unavailable to the scientific community, with only a few efforts made to build open-access repositories of breast models. Zastrow et al. [26] published a repository with nine anatomically realistic breast models derived from T1-weighted MRI images of healthy volunteers, containing seven tissue categories: glandular-high, glandular-median, glandular-low, fat-high, fat-median, fat-low, and transitional. Synthetic skin and muscle layers, with 1.5 mm and 0.5 cm thick respectively, were also added to the models. Later, Omer and Fear [44] made available a public repository of anthropomorphic breast models generated from 3.0T MRI images of healthy volunteers. The available five healthy breast models include realistic distributions of skin, fat, and fibroglandular tissue. Additionally, one diseased model containing a malignant tumor was derived from another 1.5T MRI scan and added to the repository. The tumor in this scan was segmented and made available as a separate model. Recently, a repository containing models of twenty-two breast cancer patients was made available for the development of breast cancer hyperthermia devices and treatment planning [45]. These models include anatomically accurate representations of skin, bone, fat, fibroglandular, and muscle tissues of both breasts, as well as malignant tumors.

In this paper, we introduce a new repository of anatomically realistic breast models for diagnostic and therapeutic microwave applications. Our models were derived from high-resolution T1-weighted MRI scans of patients with benign tumors and cancer patients, allowing modeling breast tumors with a high degree of realism regarding shape, dimension, location, and heterogeneity. Currently, models containing anatomical representations of skin, fat, fibroglandular, muscle, benign and malignant tumors from fifty-five patients have been made available; further efforts are being made to substantially increase the dataset. The repository offers a variety of breast models, which can be tumor-free or contain single or multiple tumors; combinations of multiple benign tumors, multiple malignant tumors, or combinations of both can be found in models with multiple tumors. It is worth noting that only six patient models include tumors in both breasts. We included breast tumors ranging in size from half centimeter to eight centimeters, exhibiting diverse shapes (smoothness) and levels of heterogeneity. This repository includes the model files, allowing an easy reconfiguration of breast tissue models, if required; and an executable file enabling the generation of models that incorporate the dielectric properties of tissues for a chosen frequency value within the range of 3-10 GHz (with a step of 0.01 GHz), which fits investigators’ needs and all developmental stages of any MWI prototype system. The developed repository can be accessed in [46].

## Materials and methods

### Dataset

The repository of anthropomorphic numerical models of the breast comprises MRI scans collected at Hospital da Luz-Lisboa between 09/25/2019 and 06/29/2022—clinical studies under references CES/44/2019/ME and CES/34/2020/ME. A written informed consent was obtained from all participants and exams were anonymized before processing. Patients were scanned in a prone position using a 3.0T MAGNETON Vida clinical magnetic resonance scanner (Siemens Healthineers, Erlangen, Germany) with a dedicated breast coil (Siemens Breast 18 coil, Siemens Healthineers, Erlangen, Germany). Two MRI sequences were used: the direct coronal isotropic three-dimensional (3D) T1-weighted (T1-w) Fast Low Angle Shot 3D (fl3D) Volumetric Interpolated Breath-hold Examination (VIBE) Dixon image sequence (T1-w Dixon), which allows obtaining four sets of images: in-phase (I), out-of-phase (O), fat-only (F), and water-only images (W); and the Dynamic Contrast Enhanced (DCE) transversal 3D T1-w Fast fl3D Spectral Attenuated Inversion Recovery (SPAIR) sequence, which consists of six sets of images—a pre-contrast image acquired before the injection of intravenous gadolinium, and five post-contrast images. Moreover, digital subtractions of each post-contrast image from the pre-contrast image were also obtained to enhance tumors and annul hypersignal regions previously present in pre-contrast images.

This repository includes scans from patients with breast tumors scored by a radiologist with BI-RADS 2 and 3, and with BI-RADS 5 and 6 [47], which were classified as benign tumors and malignant tumors, respectively. Currently, the repository includes exams of 55 patients with a total of 84 breast tumors: 46 benign and 38 malignant; new exams will be added as the processing of each exam is completed.

### Model processing

To create 3D anatomically realistic models of the breast, two MRI sequences were used for tissue segmentation. T1-w Dixon-I and T1-w Dixon-F images were used for fat, fibroglandular, skin and muscle segmentation, and a subtraction image obtained from the T1-w DCE-fl3D sequence was used to segment the breast tumors. Image registration using the Insight Toolkit (ITK) implementation (SimpleITK) [48] was performed to align and correctly superimpose the two sequences. For all images, the pre-processing pipeline included the correction of the bias field via SimpleITK N4BiasFieldCorrectionImageFilter implementation [49], followed by Minimum-Maximum data normalization [50] and a median filtering step for noise removal and edge smoothing [51, 52]. For scans with infra-centimetric tumors, the median filtering step was removed from the pre-processing pipeline, otherwise the original size and shape of the tumors would be compromised.

Data processing includes: (1) the estimation of the sternum position using the T1-w Dixon-W images; (2) the use of the sternum position as the seed for the region growing algorithm which is applied to the T1-w Dixon-F images in order to separate fat tissue of the breast region from fat tissue from the thoracic cavity; (3) the dilation of the resulting fat mask using a structuring element of radius 3 and whitening out of the anterior part of the body to include fat, skin and fibroglandular tissue, resulting in a mask of the entire breast region; (4) the dilation of the anterior and posterior part of the breast contour to obtain the final skin and muscle masks, respectively; (5) the use of a Gaussian Mixture Model to separate the fat tissue from the fibroglandular tissue using the T1-w Dixon-I images [26]. Additionally, these two categories (fat and fibroglandular) were each further subdivided into: low, medium, and high according to voxel intensity, allowing to incorporate further tissue heterogeneity. Region growing [53] and Hoshen-Kopelman [54] algorithms were applied to pre-processed subtraction images from the DCE-fl3D sequence for tumor segmentation. Details regarding breast model processing are described in [55].

### Dielectric properties assignment

The dielectric properties of segmented tissues were determined using a piecewise linear interpolation method, inspired by [26]. This approach facilitated the mapping of the T1-w Dixon-W voxel intensities to the tissue’s dielectric property curves. It is important to note that T1-w Dixon-W signal intensities are directly proportional to the amount of hydrogen nuclei in the tissues, implying that higher voxel intensities correspond to greater water content in the tissues [56].

For fat, fibroglandular, skin, and muscle tissues, the Debye parameters used for the relative permittivity and conductivity curves are well-documented in the literature [57].To account for tissue heterogeneity in our models, a 5% dielectric variation was introduced with respect to the nominal properties for skin and muscular tissue. The generated upper and lower bound curves for muscle and skin were subsequently assigned to the highest and lowest intensity voxels present in the segmented tissues, respectively. The remaining voxels within the segmented tissues were linearly mapped to a value between the curves, via a piece-wise linear strategy previously described in [26].

**Fig 1:**
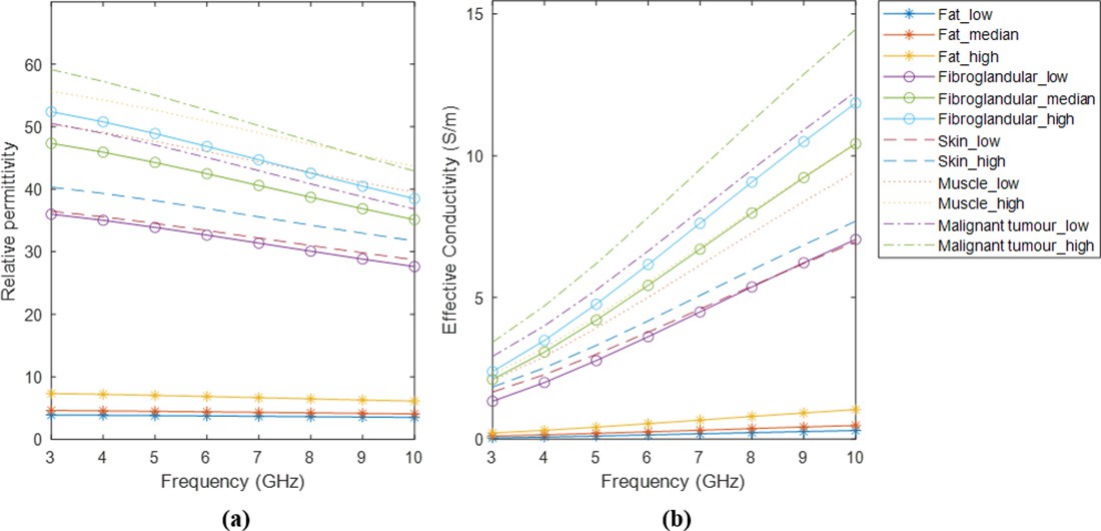
Dielectric property curves of normal breast tissues and tumors for frequencies between 3-10 GHz. (a) Relative permittivity curves. (b) Effective conductivity curves.

The 1-pole Cole-Cole parameters of the malignant tumors dielectric property curves are available in [32]. These parameters were subsequently converted into Debye parameters, as detailed in [55]. Benign breast tumors and breast tissues with low adipose content exhibit similar dielectric properties [32]. Consequently, we assumed that the dielectric properties of benign tumors fall within the range defined by the fibroglandular curves. Fig 1 illustrates the dielectric property curves for all breast tissues within the 3-10 GHz range.

### Repository

Our repository is divided into folders, one per patient. So far, we were able to populate the database with exams from 55 patients. Each folder is identified with patient ID and contains the pre-processed T1-w Dixon-W image (with 0.9965 × 0.9965 × 1 mm^3^ spatial resolution) of the patient, and two maps with labelled tissue types, identified as ‘Label_map_simple’ and ‘Label_map_detailed’. In ‘Label_map_simple’, we present a simplification of the breast, considering only benign breast tumors (label -4), malignant tumors (label -3), skin (label -2), muscle (label -1), background (label 0), and a combination of fat+fibroglandular tissue (label 1), i.e., the breast is assumed to be composed of fat, and fibroglandular tissue is disregarded. In ‘Label_map_detailed’, in addition to benign and malignant breast tumors, skin, muscle and background, we also include three sub-categories of fat and fibroglandular tissues according to voxel intensity, as well as a transitional tissue between these two tissue types in the label map, following the rationale in Zastrow et al. [26]. The new labels of the segmented tissues are the following: benign breast tumors (label -4), malignant tumors (label -3), skin (label -2), muscle (label -1), background (label 0), fibroglandular_low (label 1), fibroglandular_median (label 2), fibroglandular_high (label 3), transition (label 4), fat_low (label 5), fat_median (label 6), and fat_high (label 7). All images are available in MetaImage Medical Format MHA file format. Furthermore, information such as the Body Mass Index (BMI), and breast tumor location, number, type, and dimension per patient is given. In exams with more than one breast tumor with the same classification, tumors are referred to as ‘XS—extra small size’, ‘S—small size’, ‘M—medium size’, ‘L—large size’, and ‘XL—extra large size’ according to their relative dimensions. The tumors vary in size between approximately 0.5 cm and 8 cm.

Additionally, the repository also provides an executable file—the ‘dielectric_properties_assignment.exe’—which allows researchers to obtain the relative permittivity and effective conductivity maps of all exams for a specific frequency in the range of 3 to 10 GHz, with a step of 0.01 GHz. These dielectric property maps are available both in MHA and MATLAB file formats.

## Results and discussion

**Fig 2:**
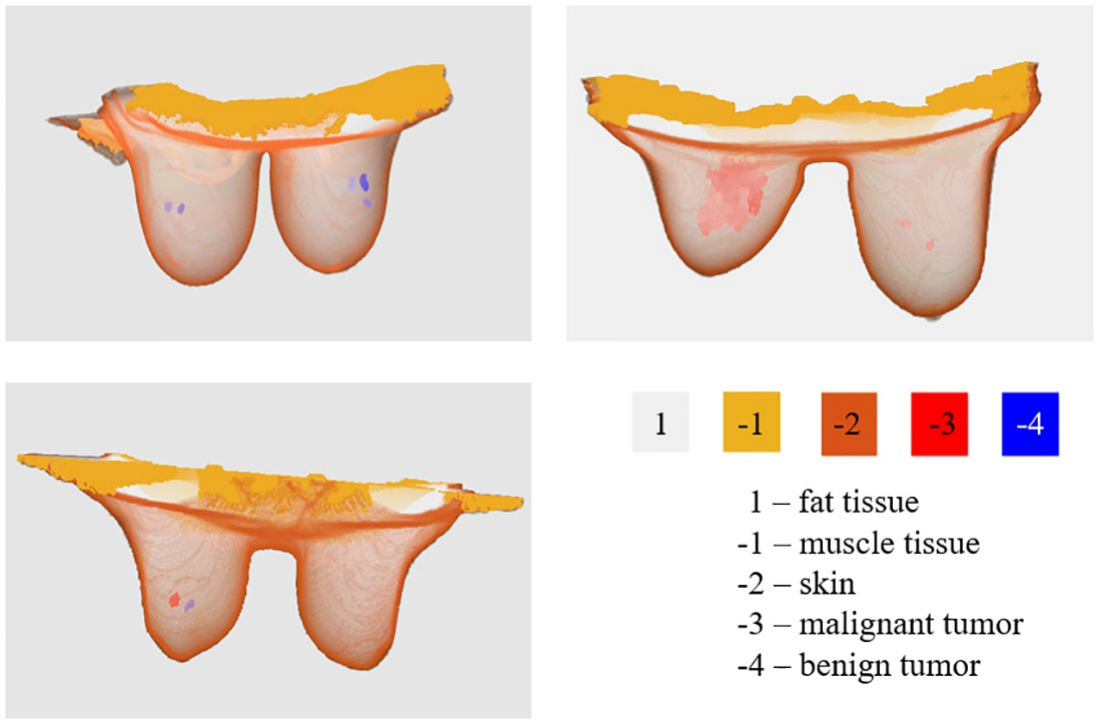
Examples of breast models with multiple tumors available in the repository. Exams 31 (top left) and 2 (top right) include only benign and malignant tumors in both breasts, respectively. Exam 4 (bottom left) exhibits a malignant and a benign tumor in one breast, and a healthy breast.

The developed repository provides bilateral models of the breast region, including benign and malignant breast tumors. Cases with multiple tumors are also available to increase the set of scenarios provided to microwave imaging studies. Fig 2 shows some examples of breast models with multiples tumors that are available in the repository [46].

**Fig 3:**
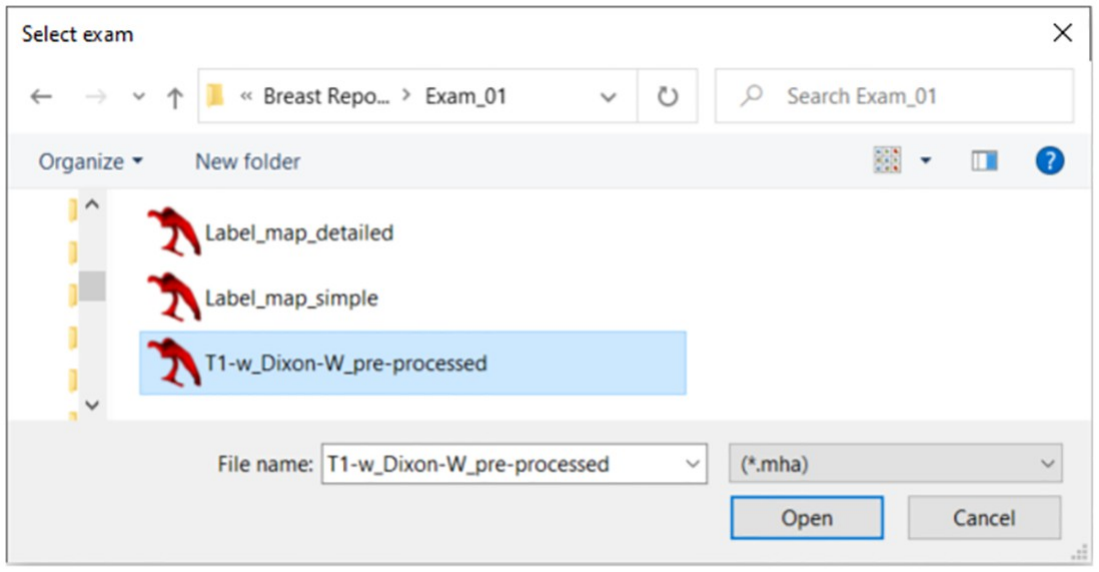
Exam selection window of the executable file.

**Fig 4:**
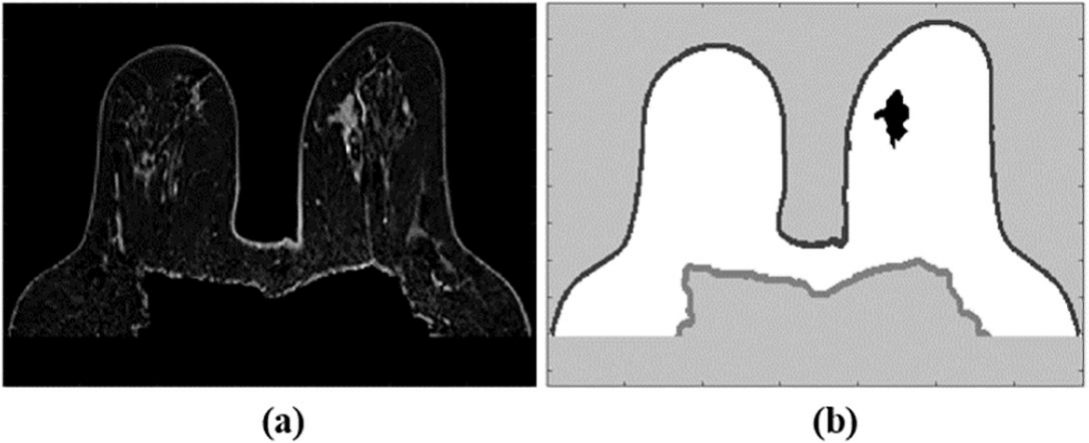
Example of a MRI exam and corresponding label map (Exam 11 of the repository). (a) Axial view of a T1-w_Dixon-W_pre-processed image. (b) Axial view of the corresponding Label_map_simple.

Good practices for the use of our repository include the download of the images and the executable file in the same directory. When opening the ‘dielectric_properties_assignment.exe’ file, the exam selection window will pop up as shown in Fig 3. Fig 4 is an example of a T1-w Dixon-W image and the corresponding simple label map.

**Fig 5:**
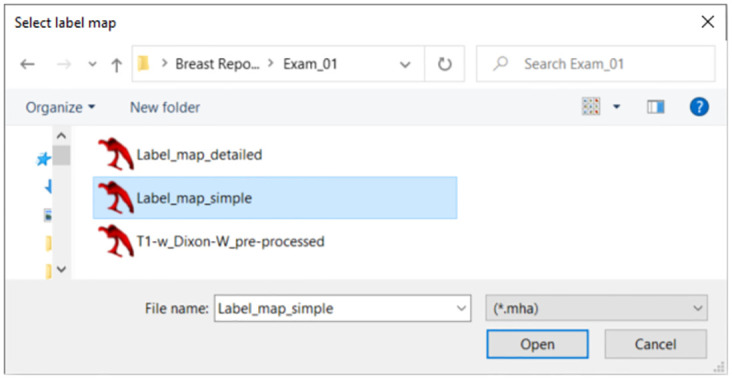
Label map selection window of the executable file.

After selecting the exam, researchers should choose the desired label map as soon as the second window pops up, as depicted in Fig 5. Please be aware that customized label maps, tailored to meet the specific requirements of researchers, can be created by modifying the labels associated with segmented tissues from the provided label maps.

**Fig 6:**
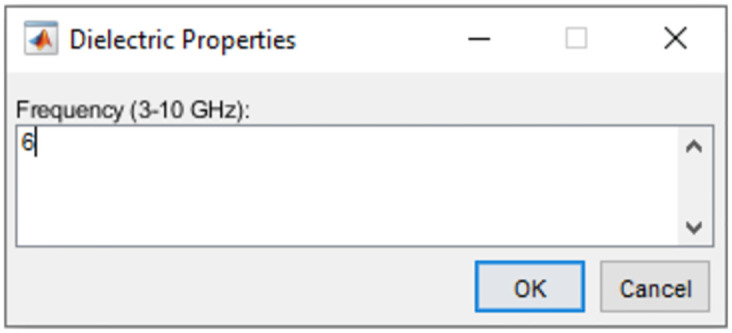
Frequency selection window of the executable file.

A frequency value between 3 and 10 GHz, with a step of 0.01 GHz can be chosen in the third pop-up window, as shown in Fig 6. This feature allows the generation of multiple numerical models containing tissue dielectric properties that match the interests of researchers in the medical MWI field.

**Fig 7:**
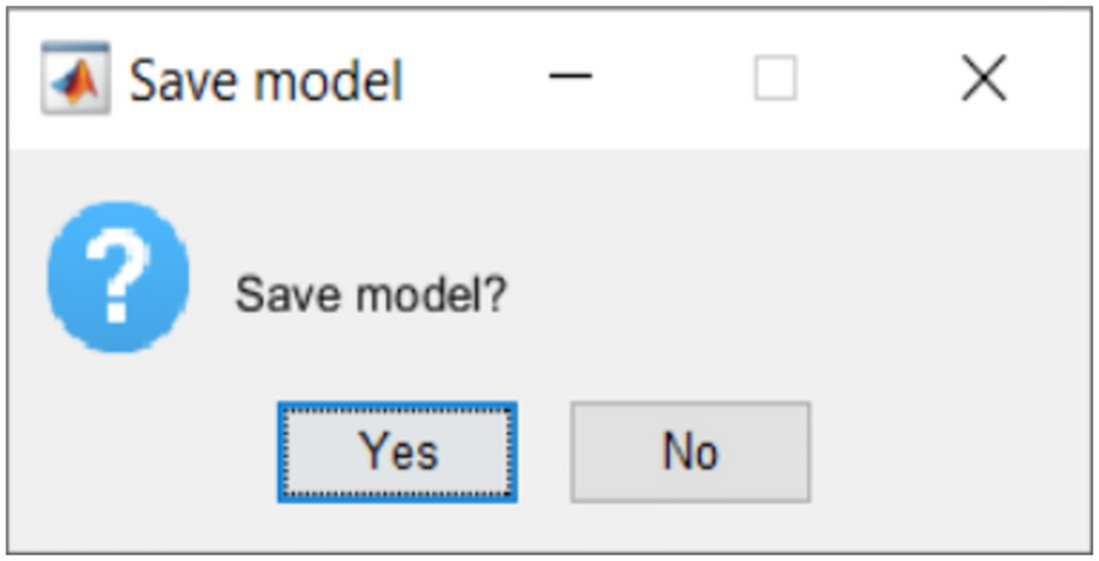
Save model window of the executable file.

Researchers can save the relative permittivity and effective conductivity maps by selecting “Yes” in the fourth pop-up window. An illustration is shown in Fig 7.

**Fig 8:**
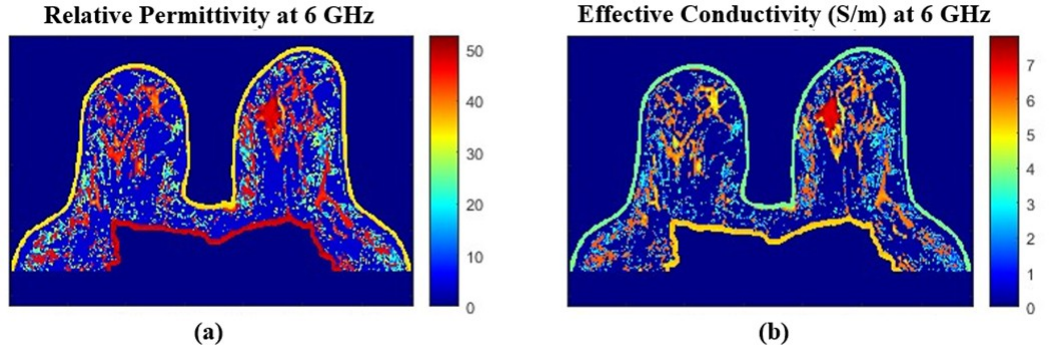
Dielectric property maps for a frequency of 6 GHz. (a) Axial view of the relative permittivity map for a frequency of 6 GHz. (b) Axial view of the effective conductivity map for a frequency of 6 GHz.

If the “Yes” button is selected in Fig 7, the maps of relative permittivity and effective conductivity will be saved in the ‘SavedFiles’ folder of that same directory and under the name Permittivity_Matrix_’freq’_’date’_’time’ and Conductivity_Matrix_’freq’_’date’_’time’, respectively. ‘freq’ stands for the chosen frequency and ‘date’ and ‘time’ are set automatically at the moment of saving. Fig 8 shows the dielectric properties maps for a frequency of 6 GHz.

## Conclusions

In this paper, we present a new open-access repository of MRI-derived numerical breast models containing normal breast tissues, as well as benign and malignant tumors, suitable for MWI purposes. Note that the available models can be utilized for alternative applications, for example MW hyperthermia, provided that the appropriate properties are attributed to the tissue maps. The patient models encompass both breasts, wherein a minimum of one breast contains at least one tumor. Dielectric property maps for frequencies between 3 and 10 GHz can be generated through an executable file available in the repository. This is also the first repository reporting benign tumors, and providing models with multiple tumors, allowing for complex and realistic test scenarios needed for feasibility and performance assessment of MWI devices. Currently, fifty-five patient models are available; further efforts are being made to substantially increase the dataset.
